# The addition of simvastatin administration to cold storage solution of explanted whole liver grafts for facing ischemia/reperfusion injury in an area with a low rate of deceased donation: a monocentric randomized controlled double-blinded phase 2 study

**DOI:** 10.1186/s12893-018-0455-7

**Published:** 2018-12-27

**Authors:** Duilio Pagano, Elisabetta Oliva, Simone Khouzam, Fabio Tuzzolino, Davide Cintorino, Sergio Li Petri, Fabrizio di Francesco, Calogero Ricotta, Pasquale Bonsignore, Alessandro Tropea, Sergio Calamia, Federico Costanzo, Angelo Luca, Salvatore Gruttadauria

**Affiliations:** 1 Department for the Treatment and Study of Abdominal Diseases and Abdominal Transplantation, IRCCS-ISMETT (Istituto di Ricovero e Cura a Carattere Scientifico - Istituto Mediterraneo per i Trapianti e Terapie ad alta specializzazione), UPMC (University of Pittsburgh Medical Center) Italy, Via E. Tricomi 5, 90127 Palermo, Italy; 2 Fondazione Ri.MED, Palermo, Italy; 3 Sidney Kimmel Medical College - Thomas Jefferson University, Philadelphia, PA USA; 4 Research Office, IRCCS-ISMETT, UPMC Italy, Palermo, Italy; 5 Department of Diagnostic and Therapeutic Services, IRCCS-ISMETT, UPMC Italy, Palermo, Italy

**Keywords:** Donor after brain death, Liver transplantation, Simvastatin, Ischemia/reperfusion injury

## Abstract

**Background:**

Liver transplantation is the best treatment for end-stage liver disease. The interruption of the blood supply to the donor liver during cold storage damages the liver, affecting how well the liver will function after transplant. The drug Simvastatin may help to protect donor livers against this damage and improve outcomes for transplant recipients. The aim of this study is to evaluate the benefits of treating the donor liver with Simvastatin compared with the standard transplant procedure.

**Patient and methods:**

We propose a prospective, double-blinded, randomized phase 2 study of 2 parallel groups of eligible adult patients. We will compare 3-month, 6-month, and 12-month graft survival after LT, in order to identify a significant relation between the two homogenous groups of LT patients. The two groups only differ by the Simvastatin or placebo administration regimen while following the same procedure, with identical surgical instruments, and medical and nursing skilled staff. To reach these goals, we determined that we needed to recruit 106 patients. This sample size achieves 90% power to detect a difference of 14.6% between the two groups survival using a one-sided binomial test.

**Discussion:**

This trial is designed to confirm the effectiveness of Simvastatin to protect healthy and steatotic livers undergoing cold storage and warm reperfusion before transplantation and to evaluate if the addition of Simvastatin translates into improved graft outcomes.

**Trial registration:**

ISRCTN27083228.

## Background

Liver transplantation (LT) is the treatment of choice for end-stage liver disease. Ischemia/reperfusion (I/R) injury during the conventional cold storage and preservation of donated livers is a key determinant of graft function after LT. Hypoxia and reperfusion activate innate mechanisms of inflammation that cause injury and underline delayed graft function. [1–3] I/R injury causes almost 10% of early organ failure and can lead to the higher incidence of both acute and chronic rejection. [4, 5] Simvastatin suppresses the inflammatory effect in vascular endothelial cells, thereby protecting the liver against I/R injury and its effects on grafts from extended-criteria donors. [6–9] Statins or HMG-CoA inhibitors were primarily designed to decrease cholesterol levels. However, they have also shown to have beneficial cholesterol-independent effects, such as up-regulating the expression of the vasoprotective transcription factor Kruppel-like factor 2 (KLF2) and consequently, its derived transcriptional programs. Simvastatin protected healthy and steatotic rat livers undergoing cold storage and warm reperfusion before transplantation. [10, 11]

While the issue of I/R injury after LT has been addressed several times and pathophysiology of this entity has been extensively investigated in the context of pre-clinical studies, the attempts to solve this clinical problem have been scarce and unstandardized. This proposal offers a more targeted method of stratifying the differences on the liver sinusoidal milieu. The differences will be obtained with neither a complex therapeutic approach of combining different pharmacologic strategies nor the usage of machine perfusion methods, but by the sole cost of a single drug/placebo administration. In this novel approach, the infusion of Simvastatin will be applied directly into the donor liver’s portal vein after donor hepatectomy. The clinical trial allows avoidance of systemic infusion, so the protocol could also be applied in cases of multiorgan deceased donor retrievals.

### Donor shortage

LT is the only life-saving therapy for most types of end-stage liver disease. In Italy, the overall rate of cadaver donation is 22.2 donors per million per year, and in some regions, like Sicily, the rate is 12.6 donors per million per year. In the past few years, IRCCS-ISMETT, a transplant hospital in Sicily, has been committed to counteracting this organ shortage. Over the years, the number of transplants has steadily increased thanks to marginal donors, living donors, and split LT. ISMETT has implemented techniques and strategies to most efficiently use valuable resources in order to maintain a high level of LT. From July 1999 till May 2014, we performed 902 LTs for adults: 755 (84%) were performed by using 601 whole livers (80%), 99 (13%) were living-related LTs, and 55 (7%) were split LTs. The one-year survival rate (cadaveric liver transplantation) is 85.4%. [12–14]

The shortage of organs led to the use of steatotic liver grafts from extended-criteria deceased brain donors (DBDs), even though these livers are more susceptible to I/R injury and have poorer outcomes than non-steatotic livers. Preclinical studies have demonstrated that the negative effects of I/R can be prevented by adding Simvastatin to the cold preservation solution shortly before procurement to protect both parenchymal and endothelial components of the liver after warm reperfusion.

The proposed project aims to be the first prospective randomized clinical trial to prove the efficacy of Simvastatin in preventing I/R injury and expand the donor pool.

### Purpose

The primary endpoint is to investigate the effect of intra-gastric Simvastatin administration using a nasogastric cannula in addition to cold-storage on 3-month, 6-month and 12-month graft survival after LT. As secondary endpoints, we will compare 3-month, 6-month and 12-month patient survival, hospitalization stays, and all transplant-related complications in terms of the multi-tier Clavien grading system after LT. This will allow us to identify a significant relation into two homogenous groups of liver transplanted patients, only differing for Simvastatin or non-administration regimen; using the same procedure, with identical surgical instruments, and medical and nursing skilled staff.

## Methods

### Trial design

The trial will employ a randomized administration of a single oral dose of 80 mg of Simvastatin (Sandoz®, Holzkirchen, Germany) (Group 1) or placebo (Group 2) per adult cadaveric donor through a nasogastric tube 1 h before the organ procurement, to investigate the effect of these interventions on graft survival after LT.

### Setting

The trial will be conducted in the operating rooms of a private hospital in Palermo (ISMETT), Italy and in every external hospital where all of the eligible DBDs are located. ISMETT was developed specifically for transplantation through a partnership of the Sicilian regional government and the University of Pittsburgh Medical Center. It is the first hospital in Southern Italy certified by Joint Commission International for excellence in clinical practice and is one of the first hospitals in Italy to use an entirely computerized process. It includes laboratories for clinical chemistry, microbiology, immunology and pathology, as well as laboratories that are equipped to support research activities. Liver transplantation is at the core of ISMETT transplantation programs. The ISMETT Abdominal Surgery and Transplantation operational unit has a team of 6 expert attending physicians and 2 surgery fellows, who manages the selection and care of patients requiring LT both from cadaveric and living donor. In ISMETT there is close collaboration and daily interdisciplinary teamwork with colleagues from other disciplines at ISMETT, including hepatologists, critical care clinicians, radiologists, endoscopists, and researchers from Ri.MED Foundation. An intense daily collaboration is performed with nurses, physical and respiratory therapists. One skilled research nurse will be recruited for guaranteeing the correct schedule of outpatient visit examinations, patient appointments and maximize the adherence. Ri.MED investigator will coordinate the effective functioning of the data and safety monitoring board (DSMB), maintaining information reviewed, discussed and recorded with the strict confidentiality. In addition, she will ensure the correct data recording of retrieval procedures, patient enrolment and data analysis in agreement DSMB recommendations and respect of the entire study phases.

### Eligibility criteria

Participants will be deemed eligible for the trial if they are 1) patients (male and female) over 18 years of age, 2) deceased brain donors (male and female) over 18 years of age, 3) female patients who are of child-bearing potential must ensure effective methods of birth control during the first 6 months of study following the whole LT, 4) patients with end-stage liver disease or terminal cancer liver disease, and a suitable candidate are placed on waiting list for primary LT at IRCCS-ISMETT, 5) patients who underwent liver transplantation from deceased brain donors (not identical human leukocyte antigen - HLA) compatible in terms of blood group ABO, 6) patients who can understand the purpose and risks of the study, have been fully informed and given written informed consent, 7) patients who are unable to read and/or write, but are fully capable of understanding the oral proposal by the researcher (or the appointed representative) and have given oral informed consent that is testified in writing by an independent third person.

The following subjects will be excluded by donor population: 1) pregnant or breast-feeding donors, 2) donor with autoimmune disease or allergies to statins, 3) donors requiring ongoing dosing with a systemic immunosuppressive drug at time of harvesting procedures, 4) donors known to be human immunodeficiency virus (HIV) positive, 5) donors with malignancy or history of malignancy, except for non-metastatic basal or squamous cell carcinoma of the skin that has been treated successfully, 6) donors had previously received or is receiving an organ transplant.

The following subjects will be excluded by recipient population: 1) recipients with acute liver disease, 2) pediatric patients or pediatric donors, 3) patients undergoing liver re-transplantation, 4) patients undergoing split or living donor liver transplantation, 5) patients undergoing combined liver- or other solid (abdominal or thoracic) organ(s) transplantation, 6) patient who is participating or have been participated to another clinical trial/study and/or has consumed an experimental drug in the last 30 days, 7) any clinically relevant conditions that might affect study participation and/or study results, 8) patients with history of allergy or intolerance to statins, 9) patients with history of substance abuse, and/or psychiatric disorders or conditions that may invalidate communication due to their inability to understand the potential risks and benefits of the study, 10) patients greater than 65 years old.

### Interventions

**Fig. 1:**
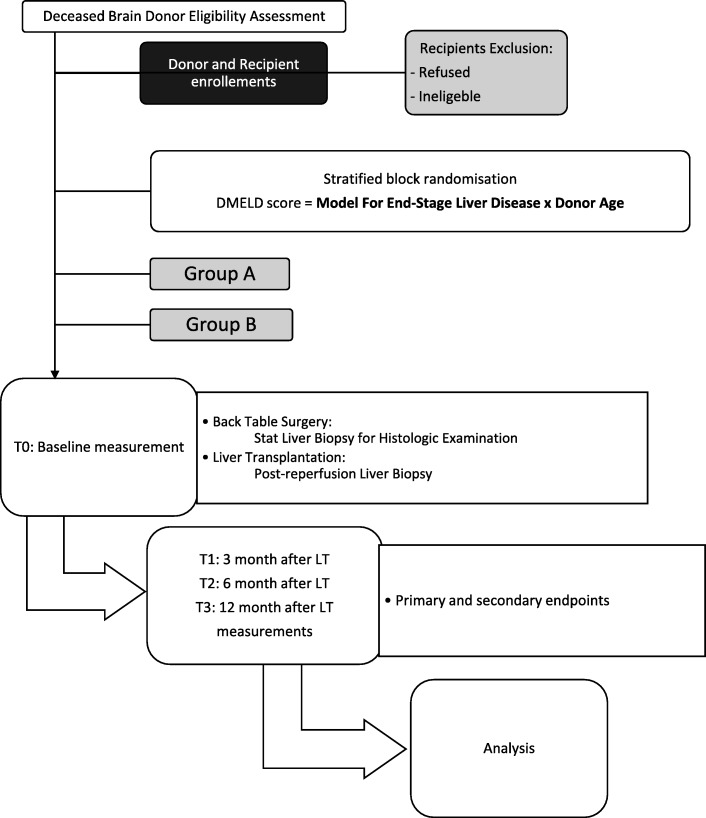
Study flow diagram

Participants will be allocated to one of two groups (see Fig. 1): Group one will receive an organ procured in a DBD after preoperative administration of Simvastatin; group two will comprise the control group receiving no DBD intervention before the standard organ procurement in a DBD or after.

#### Experimental treatment

Simvastatin tablets on the market will be pulverized and placed in capsules in order to then be emptied at the time of preparation of the suspension at the clinical site during the start of the cold phase of the collection. An Investigational Medicinal Product Dossier (IMPD) will be drawn up before the trial begins and the authorization of the National Competent Authority (NCA) has been granted. The drug will be administered in the deceased donor prior to the allograft procurement by using a nasogastric tube, and it will be subsequently transplanted into the recipient. Two Simvastatin 40 mg tablets (Sandoz®, Holzkirchen, Germany) will be pulverized and encapsulated in order to be emptied at the moment of the preparation into a hydro-alcoholic suspension at the clinical site during the start of the cold phase collection. The suspension will then be administered via an intragastric route using a nasogastric cannula one hour before vascular clamping in the DBD surgical liver procurement procedures. [15]

Criteria for discontinuing or modifying allocated interventions for a given trial participant will be defined after histologic examinations of back-table and post-reperfusion liver biopsies (Fig. 1). The therapeutic intervention will be applied to the DBD before transplantation, and for this reason, no strategies to improve adherence to intervention protocols, and any procedures for monitoring adherence will be implemented. No relevant concomitant care and interventions are prohibited during the trial.

#### Usual care

Patients in group two will receive ‘usual care’ only. Placebo will include the aforementioned preoperative administration of 2 hard jelly capsules (dimension = 0), containing an orange-swedish microcrystalline cellulose.

### Outcomes

The primary outcome measure in this trial is postoperative graft survival after LT. The secondary outcomes of interest are patient survival, hospitalization stays, and all transplant-related complications in terms of the multi-tier Clavien grading system after LT. The grading system is used in order to identify a significant relation into two homogenous groups of liver transplanted patients, which only differ for Simvastatin or non-administration regimen utilizing the same procedure, with identical surgical instruments, and medical and nursing skilled staff. Baseline data on graft function and patient performance status will be collected immediately prior to discharge from the recovery unit (T0). Follow-up measures will be collected at three time points**:** 3-month (T1), 6-month (T2) and 12-month (T3)**,** after LT (see Fig. 1). All participants will be routinely evaluated with a post-reperfusion liver biopsy (sample), intensive care monitoring, daily laboratory and ultrasound examinations for the first week after the operation and when required during their hospital stay.

### Sample size

We hypothesize that Simvastatin’s protective effects may occur in the liver during the harvesting procedure from the deceased donor and could significantly ameliorate post-LT outcomes. To reach the above-mentioned goals, we decided to recruit 106 patients. This sample size achieves 90% power to detect a difference of 14.6% between the two groups survival using a one-sided binomial test. The target significance level is 0.05. These results assume a null hypothesis that the proportion of the group with the standard procedure is 0.85.

### Interim analysis

Two interim analyses of drug tolerance and efficacy will be performed 6 months after the trial enrollment starting, and when 75% of participants have been enrolled in the trial, respectively. The level of significance will maintain an overall *P* value of 0.05 and be calculated according to the O’Brien-Fleming stopping boundaries. [16]

The DSMB will be instituted as an independent group of experts that will advise the clinicians and the study investigators, without direct involvement in the conduct of the study, nor financial, proprietary, professional, or other interests that may affect impartial, independent decision-making. The ISMETT Director of the Department for the Treatment and Study of Abdominal Diseases and Abdominal, the ISMETT Medical and Scientific Director and the primary investigator (PI) will have primary responsibility for the DSMB formation and for identifying the DSMB Chair and the terms of membership for the entire duration of the study. The DSMB will deliberate processes, including event triggers that would call for an unscheduled review, stopping guidelines, unmasking (unblinding) and voting procedures prior to initiating any data review. ISMETT investigators will collect, assess, report, and manage solicited and spontaneously reported adverse events and other unintended effects of trial interventions or trial conduct.

### Randomization

A statistician group, with no clinical involvement in the trial, will computer-generate a stratified block randomized sequence, based on D-MELD score [17–19]. This list will remain concealed from the trial coordinator at all times. When a DBD becomes available, the trial coordinator will assess the previously enrolled participant, which was considered suitable for the above-mentioned DBD [19]. S/he will obtain the treatment allocation by a specific website, dedicated for electronic clinical report form depository (https://simvalt.fullcro.org/). S/he will then inform the appropriate donor nursing and medical staff who will deliver the intervention(s). The trial coordinator will not collect outcome data, deliver the intervention(s), or provide patient care.

### Blinding

Outcome measures will be collected by recovery unit medical and nursing staff that are blinded to the participants’ treatment allocation. The interventions will be delivered by separate groups of operative staff (operative donor team), and each group will be blinded to the treatment delivered by the other. The trial will adhere to procedures to maintain separation between the data management staff who will record the outcome data and the donor operative nursing and medical staff who will deliver the intervention(s).

### Data collection

The data from patients enrolled in the Simvalt study are collected by EDC (electronic data capture) using a web-based eCRF. The link to access to eCRF is https://simvalt.fullcro.org, the data, once entered in eCRF, are stored in a database. The credentials (user name and password) to access to eCRF are given individually to each user upon request of the Sponsor, after the request the credentials are provided within 1 working day; the password respects the security requirements (it must have at least one capital letter, one number, one special character and it must be composed of at least ten digits). The patient data are anonymized; it means that each patient is identified with a code which is automatically generated by the eCRF at the moment of enrolment, in the eCRF only the year of birth and the gender of patient is collected, no other personal data (name, initials, complete date of birth) are collected, so only the Investigator at the site can know the identity of the patient.

Collection of laboratory evaluations, and storage of biological specimens (liver biopsies) for genetic or molecular analysis in the current trial and for future use in ancillary studies, will be stored at ISMETT.

#### Statistical analysis

Intention-to-treat analysis will be applied. Quantitative variables will be compared with the Student t-test or the Wilcoxon rank-sum test and categorical variables will be compared with the chi-squared test or Fisher’s exact test as appropriate. No a-priori criteria will be set for an early end of the study. Data will be analyzed according to the 2 × 2 randomized factorial study design. All of the phases will follow the Consolidated Standards of Reporting Trials 2010 Statement to improve the reporting of parallel-group randomized controlled trial, enabling readers to understand the trial’s design, conduct, analysis. and interpretation as well as to assess the validity of its results. It will be achieved through complete transparency from study investigators. All analyses and graphics were performed in the R statistical computing environment, version 3.4.3.

### Monitoring

Circumstances under which unblinding is permissible are related to severe and not expectable adverse recipient reaction to Simvastatin or Placebo, and PI will be involved procedure for revealing a participant’s allocated intervention during the trial. Outcome measures will be collected by recovery unit medical and nursing staff that are blinded to the participants’ treatment allocation. The interventions will be delivered by separate groups of operative staff (operative donor team), and each group will be blinded to the treatment delivered by the other. The trial will adhere to procedures to maintain separation between the data management staff who will record the outcome data and the donor operative nursing and medical staff who will deliver the intervention(s). In addition, DSMB will review the following items: 1) interim/cumulative data for evidence of study-related adverse events; 2) data quality, completeness, and timeliness; 3) adequacy of compliance with goals for recruitment and retention; 4) adherence to the protocol; 5) factors that might affect the study outcome or compromise the confidentiality of the trial data.

DSMB will provide official communications that will occur between EC and IRB, including: 1) modifications of the study protocol based upon the review of the safety data; 2) suspension or early termination of the study because of serious concerns about subjects¿ safety, inadequate performance or rate of enrolment or because study objectives have not been obtained according to pre-established statistical guidelines; 3) optional approaches for clinicians and study investigators to consider when the DSMB determines that the incidence of primary study outcomes is substantially less than expected.

### Ethical considerations

The project has been approved by the hospital’s human research ethics committee. Informed consent will be obtained from all participants. ISMETT PI and co-investigators will obtain informed consent or assent from potential trial participants or authorized surrogates, during outpatient clinic visits in waiting-list time and during the ISMETT admission date for liver transplantation.

## Discussion

This trial is the first to rigorously evaluate the effect of preoperative DBD treatment with the administration of Simvastatin on LT outcomes. The use of Simvastatin could reduce donor liver damage and decrease the length of hospital stay and the rate of complications. There is a remote probability of liver damage with the use of statins. However, Simvastatin is a well-known drug with a minimal reported risk of liver damage compared with the other statins. The factorial design of the trial enables a head-to-head comparison of the individual and cumulative effects of this intervention, which should provide valuable evidence to inform clinical practice.

### Trial status

The proposed trial has been validated and accepted for financial support by the Italian National Health Ministry (Programme of “Ricerca Finalizzata 2013” - Clinical health care research - GR-2013-02357764). Patient inclusions have been ongoing since 30 June 2018.

## Abbreviations

DBD: Donor after brain death
DSMB: Data and safety monitoring board
HIV: Human immunodeficiency virus
HLA: Human leukocyte antigen
IMPD: Investigational medicinal product dossier
KLF2: Kruppel-like factor 2
LT: Liver transplantation
NCA: National Competent Authority
PI: Primary investigator
